# A lightweight CNN for colon cancer tissue classification and visualization

**DOI:** 10.3389/fonc.2025.1659010

**Published:** 2025-10-17

**Authors:** Jie Li, Weiwei Goh, Noor Zaman Jhanjhi

**Affiliations:** Digital Health and Medical Advancement Impact Lab, School of Computer Science, Taylor’s University, Subang Jaya, Malaysia

**Keywords:** colon cancer, CNN, data cleaning, image processing, medical imaging, histopathology, lightweight model

## Abstract

**Introduction:**

Colon cancer (CC) image classification plays a key role in the diagnostic process in clinical contexts, especially as computational medical solutions become the trend for future radiology and pathology practices. This study presents a novel lightweight Convolutional Neural Network (CNN) model designed with effective data cleaning strategy for the classification and visualization of histopathology images of various colon cancer tissues.

**Methods:**

Addressing the critical need for efficient diagnostic tools in colon cancer detection, the proposed model leverages a non-pretrained architecture optimized for performance in resource-constrained environments. Utilizing the NCT-CRC-HE-100K and CRC-VAL-HE-7K datasets, this model employs a parametric Gaussian distribution-based data cleaning approach to enhance data quality by removing outliers.

**Results:**

With a total of 4,414,217 parameters and a total size of 16.9 megabytes, the model achieves a test accuracy of 0.990 ± 0.003 with 95% level of confidence, which demonstrates high precision, recall, specificity, and F1 scores across various tissue classes.

**Discussion:**

Comparative analysis with benchmark studies underscores the model’s effectiveness, while discussions on underfitting and overfitting provide insights into potential fine-tuning strategies. This research presents a robust, lightweight solution for colon cancer histopathology image classification, offering a foundation for future advancements in colon cancer diagnostics with result visualization.

## 1 Introduction

Colon Cancer (CC) has become the second leading cause of cancer deaths worldwide in the last decade. Meanwhile, the implementation of Artificial Intelligence (AI) in CC diagnostic tools has significantly boosted its accuracy and speed. Histopathology images have been widely used as a diagnostic tool to detect CC in clinical settings. Over the past years, the use of AI has boosted histopathological detections and classification tasks in different types of cancers including breast (1, 2), brain (3), lung (4), and skin (5) etc. In this case, the use of AI in histopathology image recognition has technologically revolutionized cancer diagnostics in the medical industry.

Classifying CC tissue in the diagnostic process is crucial to accurately identify and differentiate between various pathological conditions (6). In histopathology, distinguishing the tissue types such as ADI (Adipose), TUM (Tumor), MUC (Mucin), STR (Stroma), and others enables clinicians to pinpoint abnormal growth patterns, assess tumor aggressiveness, and make informed decisions about treatment strategies. AI-based classification of histopathology images could potentially be crucial for early cancer detection with accurate diagnostics in clinical practices. By automating tissue classification. Using a confusion matrix derived from a sorting model’s predictions, medical professionals gain statistical insights into both the accuracy of the diagnoses and the areas prone to error (misclassifications) (6). Thus, a clear and reliable classification solution helps refine diagnostic protocols, reduces manual errors, and potentially accelerates the turnaround time for pathology reports, ultimately leading to better patient outcomes in later treatment and therapy processes.

Deep Learning (DL) excels at advanced image recognition applications. In recent years, the field of medical image classification, particularly for CC, has seen significant advancements with the use of Convolutional Neural Networks (CNNs) in DL. However, there is still potential to improve the accuracy of AI-based medical image classification to ensure better diagnostic precision. In recent years, Dense-Net (7), Res-Net (8), and Inception V3 (9) were the most widely-used pretrained models to classify NCT-CRC-HE-100K and CRC-VAL-HE-7K datasets. While pretrained CNN models are preferred for the classification of CC tissues for diagnostic purposes, non-pretrained models possess better flexibility for task-specific goals. In addition to model generalizability, previous studies on CC tissue image classification only focused on a single dataset, yet without specification on data cleaning method on training, validation, and testing process in their CNN models. Furthermore, integration of result visualization improves intepretability of results in clinical practices. The use of lightweight models to classify CC tissues would benefit the pathological decision in the diagnosis of CC. A lightweight model solution would be more advantageous in terms of the deployment and energy consumption of front-end diagnostic devices (10).

In this case, the research objectives are: (1) to develop and finetune a lightweight non-pre-trained CNN model to classify CC histopathology images from the NCT-CRC-HE-100K and CRC-VAL-HE-7K datasets (2) to evaluate its performance compared to benchmark studies over the past 5 years, and (3) to visualize image from result of different CC tissue classes. The motivation of this research is to improve computational efficiency of AI model in clinical settings via a lightweight CNN architecture configuration with adoption of statistical data cleaning method. As a supportive diagnostic tool, visualization of result could also benefit clinician and pathologist on the final decision making to diagnosis.

## 2 Literature review

### 2.1 CNNs in histopathology analysis

The DL approaches include hybrid learning, end-to-end learning, transfer learning, explainable AI, and sampling-based learning (11). Before the use of CNN classification, Toraman et al. (12) proposed the use of an ANN model in 2019 to predict the presence of colon cancer tissue using FTIR signals on 30 colon cancer patients and 40 healthy humans, which managed to reach an accuracy of 0.957. In recent years, most medical image processing and imaging applications are powered by CNNs. Their architecture includes data preprocessing and preparation, data augmentation, feature extraction, and finally feature classification using linear or nonlinear activation functions (e.g., ReLU, GeLU, etc). CNN’s current contributions to histopathology analysis have had a profound impact on the detection of cancers such as breast (13, 14), lung (15, 16), and brain (17, 18). The most commonly-used models in the diagnosis process are based on Transformer-based and hybrid CNN-Transformer architectures for classification task.

In **Table 1**, over the past 5 years, in 2020, a study proposed a hybrid model (19) that adopted MFF-CNN with Shearlet transform for the selection of histopathology images and reached a model accuracy of 0.960 using National Cancer Center of Heidelberg and Medical Center of Heidelberg University in Germany. Bukhari et al. (20) applied ResNet 18, 30, and 50 to colonic tissue image classification, and the models reach an accuracy of up to 0.939. The accuracy of the prediction model showed a significant improvement, as shown in research by Tasnim et al. (10), where their MobileNetV2 model showed 0.997 accuracy in colon cell image classification. In the same year, Hamida et al. (21) tested AlexNet, Visual Geometry Group (VGG), and ResNet models on colon cancer histopathology images, which reached the highest accuracy of up to 0.970. Moreover, Ghosh et al. (22)designed and developed an ensemble learning method CNN model to classify CC histopathology images from the NCTCRC-HE-100K and/or the CRC-VAL-HE-7K dataset in 2021 that was able to display a 0.961 diagnostic accuracy. In the same year, Shawesh and Chen (23) applied the ResNet 50 model to classify colorectal cancer tissue histopathology images that reached 0.977 accuracy. The model was later improved by Tsai and Tao (24) to an accuracy of 0.993. Anju and Vimala (9) achieved this in 2022 by applying the InceptionV3 model to classify images of colon cancer tissue and reached a model accuracy of 0.974 using the same NCT-CRCHE-100K data set. Sakr et al. (25) designed and developed a CNN model that improved the model’s accuracy to 0.995, while a study (26) utilized a deep convolutional neural network (DCNN) model to further improve cancer recognition accuracy to 0.998. This is followed by an innovative graph-based sparse principal component analysis (GS-PCA) network model (27) detecting colon cancer tissues using histopathology images with 0.909 accuracy.

**Table 1:** Summary of recent deep learning models for colorectal cancer histopathology image classification (2020–2025).

Benchmark	Model/method	Dataset	ACC
Liang et al. (19)	MFF-CNN Shearlet Transform	NCCH/CHU	0.960
Bukhari et al. (20)	ResNet-18/30/50	CRAG	0.939
Tasnim et al. (10)	MobileNetV2	LC25000	0.997
Hamida et al. (21).	AlexNet, VGG, ResNet	Annotated WSI data	0.970
Ghosh et al. (22)	Ensemble CNN	NCT-100K/CRC-7K	0.961
Shawesh and Chen (23)	ResNet-50	NCT-100K/CRC-7K	0.977
Tsai and Tao (24)	Improved ResNet-50	NCT-100K	0.993
Anju and Vimala (9)	InceptionV3	NCT-100K	0.974
Sakr et al. (25)	Custom CNN	LC25000	0.995
Hasan et al. (26)	Deep CNN (DCNN)	LC25000	0.998
Ram et al. (27)	GS-PCA Network	Stefanie Galban Lab	0.909
Jiang et al. (28)	Multi-scale Gradient GAN	NCT-100K	0.869
Kumar et al. (29)	CRCCN-Net	NCT-100K	0.992
Fadafen and Rezaee (8)	dResNet	NCT-100K	0.997
Reis and Turk (7)	DenseNet-169	MNIST	0.950
Azar et al. (30)	Swim Transform, Color-CADx	NCT-100K	0.993
Haq et al. (31)	ResNet-18/50	Not Specified	0.987
Alzubaidi et al. (32)	ResNet-110	Warwick-QU	0.996
Pacal and Attallah (33)	ResMLP+SwimTran+Xception	NCT-100K/Kather-5K	0.991
Venkatachalam and Shah (34)	ResNet-based	LC25000	0.989
Hosny et al. (35)	Hybrid Res-Net & Inception	LC25000	0.997

Then, in 2023, Jiang et al. (28) designed a CNN model using a multi-scale gradient generative adversarial network and recorded a result accuracy of 0.869. Later in the same year, a CRCCN-Net architecture (29) using the same dataset, which obtained a high classification accuracy of 0.992. Following that, the use of the dResNet and DeepSVM methods (8) in the classification of colorectal cancer histology images. Their results suggested that the dResNet model that was trained on NCT-CRC-HE-100K had an accuracy approaching 0.997. Subsequently, Reis and Turk (7) applied DenseNet 169 architecture on the colorectal histology MNIST dataset and managed to reach a 0.950 validation accuracy. The CNN model (30) was optimised using colon cancer datasets, which improved the model accuracy up to 0.996. In 2024, a study by Sharkas and Attallah (36) applied the Swim Transform and Color-CADx approaches to their CNN models, and their results showed 0.993 accuracy in model performance. Haq et al. (31) proposed Res-Net 18 and 50 achieved 0.987 and 0.967 accuracy. Alzubaidi et al. (32) came up with the solution based on 110 variant of Res-Net architecture and achieved 0.996 accuracy. In 2025, Pacal and Attallah (33) implemented hybrid architecture and achieved (0.991 - 0.999) accuracy. Venkatachalam and Shah (34) proposed another Res-Net based architecture and gained 0.989 accuracy in a classification task. Hosny et al. (35) proposed a hybrid Res-Net and Inception based learning model for the CC classification task with 0.997 accuracy.

### 2.2 Advantages of lightweight CNN architecture

A lightweight nature is crucial for a CNN model that is developed for medical diagnoses utilizing histopathology images (10). Real-time prediction is required when it comes to medical applications using the model, particularly in situations where pathologists require a prompt and precise diagnosis (37). Lightweight CNN models exhibit computational efficiency and yield expedited predictions, being capable of operating on low-powered hardware commonly seen in medical equipment or systems such as mobile health applications and embedded devices Reddy and Dhuli (38). The size of the lightweight model restricts lightweight CNNs to have fewer model parameters and be under 20 megabytes. This is crucial for democratizing healthcare, as not all medical facilities have access to medical equipment or systems with advanced GPUs Momin et al. (39). Lightweight CNN models can be utilized in remote locations or resource-limited settings where access to extensive computing hardware is restricted (40). In low-resource environments, healthcare professionals can utilize mobile or portable diagnostic instruments equipped with lightweight convolutional neural network models to identify colon cancer (10). Lightweight models, due to their diminished parameter count, have a lower susceptibility to overfitting, particularly when trained on fewer datasets, which is a common scenario in medical picture analysis (41). histopathology image datasets for colon cancer may not be as extensive as those for general image classification, hence lightweight models facilitate improved generalization without overfitting to the training data under a limited resource.

## 3 Method

### 3.1 Data collection and preprocessing

**Figure 1:**
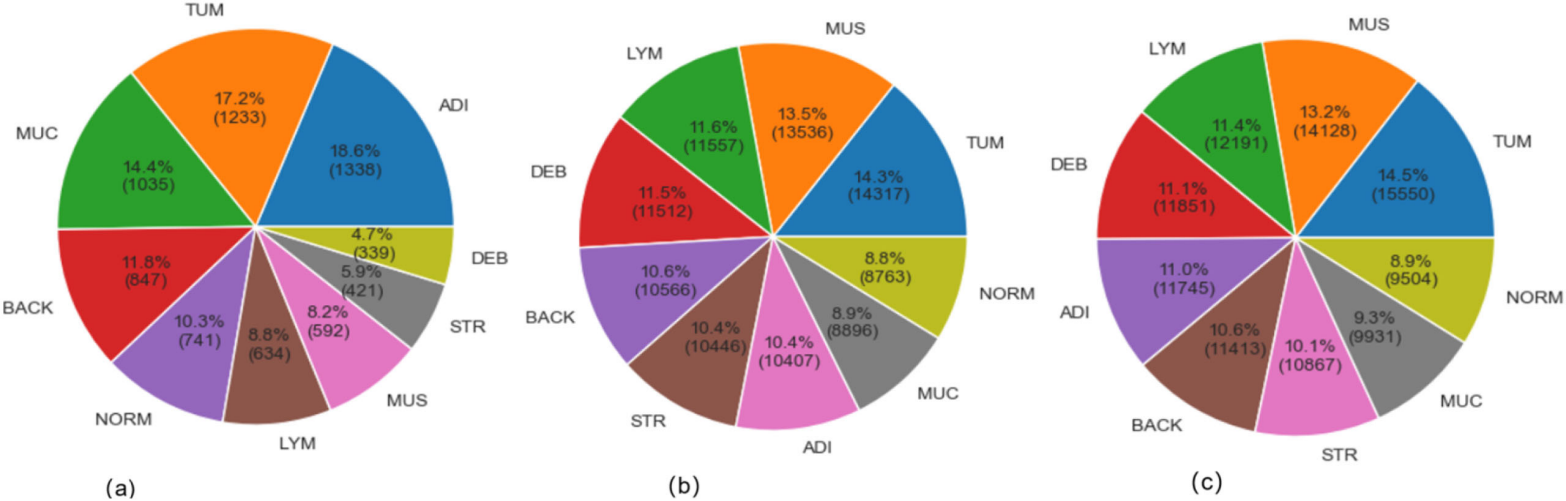
Sample distribution **(A)** CRC-VAL-HE-7K; **(B)** NCT-CRC-HE-100K; **(C)** Merged dataset.

The CRC-VAL-HE-7K (in **Figure 1A**), NCT-CRC-HE-100K (in **Figure 1B**), and their merged (in **Figure 1C**) datasets were used to train, validate, and test the proposed model. The data-splitting strategy followed the 80–20 distribution rule. In these datasets, the classified categories are ADI (adipose tissue, consisting of adipocytes), BACK (background of histopathology images), DEB (debris, useful for diagnosis of cancer), LYM (lymphocytes, cells of lymphatic system), MUC (mucus, protective layer on tissue), MUS (smooth muscle), NORM (normal tissue of colon), STR (stroma tissue associated with cancer), and TUM (epithelium tissues of adenocarcinoma).

The **Figure 1** exhibited the data distribution of the histopathology image classes in the VAL-HE-7K, NCT-CRC-HE-100K, and the merged datasets. In VAL-HE-7K, there were a total of 7,180 images where ADI has the highest number of samples (n = 1,338: 18.6%) followed by the TUM category (n = 1,233: 17.2%). This is followed by the MUC category, which has (n = 1,035; 14.4%) images. These 3 categories made up more than 50% of the dataset, and the rest of the categories — DEB, STR, MUS, LYM, NORM, and BACK— covered 4.7%, 5.9%, 8.2%, 8.8%, 10.3%, and 11.6% of the dataset, respectively. There were 100,000 histopathology images in NCT-CRC-HE100K, where TUM has the highest number of samples (n = 14,317: 14.3%), followed by MUS at n =13,536; 13.5%. LYM and DEB covered n = 11,557 (11.6%) and n = 11,512 (11.5%) of the dataset, respectively. These 4 categories made up for over 50% of the sample size, while the rest of the classes, including BACK, STR, ADI, MUC, and NORM, covered 10.6%, 10.4%, 10.4%, 8.9%, and 6.6% of the total sample size, respectively. In the combined dataset, TUM has the highest number of samples (n = 15,550: 14.5%), followed by MUS at n = 14,128: 13.2%. This is followed by the LYM and DEB categories at n = 12191 (11.4%) and n = 11,851 (11.1%). These 4 categories take up more than 50% of the total sample, and the rest of the categories, including BACK, STR, ADI, MUC, and NORM, make up 10.6%, 10.1%, 11.0%, 9.3%, and 8.9% of the total sample size, respectively. After merging NCT-CRCHE-100K and VAL-HE-7K, both the quantity and variety of the merged dataset increased.

The descriptive result in **Figure 1** detailed the class imbalance inherent in the original datasets. This research adopted strategy using weighted loss functions and data augmentation during training, rather than aggressive pre-balancing which could discard valuable data. The merged dataset exhibits a natural class imbalance, as shown in **Figure 1C**. To mitigate bias towards majority classes, we employed a dual strategy during training: (1) Class-weighted loss function: The categorical cross-entropy loss was weighted inversely proportional to class frequencies to penalize misclassifications on underrepresented classes more heavily. (2) Targeted augmentation: During training, real-time data augmentation (including rotation (± 15°), horizontal/vertical flips, and slight brightness adjustments) was applied, which artificially increases the diversity of the training set and improves generalization. All input images were normalized to the [0, 1] range based on the RGB channel means and standard deviations calculated from the merged set.

### 3.2 Data cleaning

As the key part of data preprocessing strategy, the parametric Gaussian distribution, the Equation 1, was applied based on the color distribution test, where input *x* was normalized based on the mean value (*µ*) and standard deviation (*σ*) as the applied data cleaning method.


(1) $$f(x|\mu ,\sigma^{2})=\frac{1}{\sqrt{\left(2\pi \sigma^{2}\right)}}Exp(-\frac{{\left(x-\mu \right)}^{2}}{\left(2\sigma^{2}\right)})$$

and Equation 2 allocates *x* within the range of the 99% level of distribution:


(2) x∈[μ±2.576σ]

The Gaussian (normal) distribution is a fundamental concept in statistics. Many natural and measurement-related phenomena, including pixel intensities and feature distributions in images, tend to follow a normal distribution, especially after normalization or standardization for a consistent and concentrated sample distribution.

### 3.3 Model design and development

**Figure 2:**
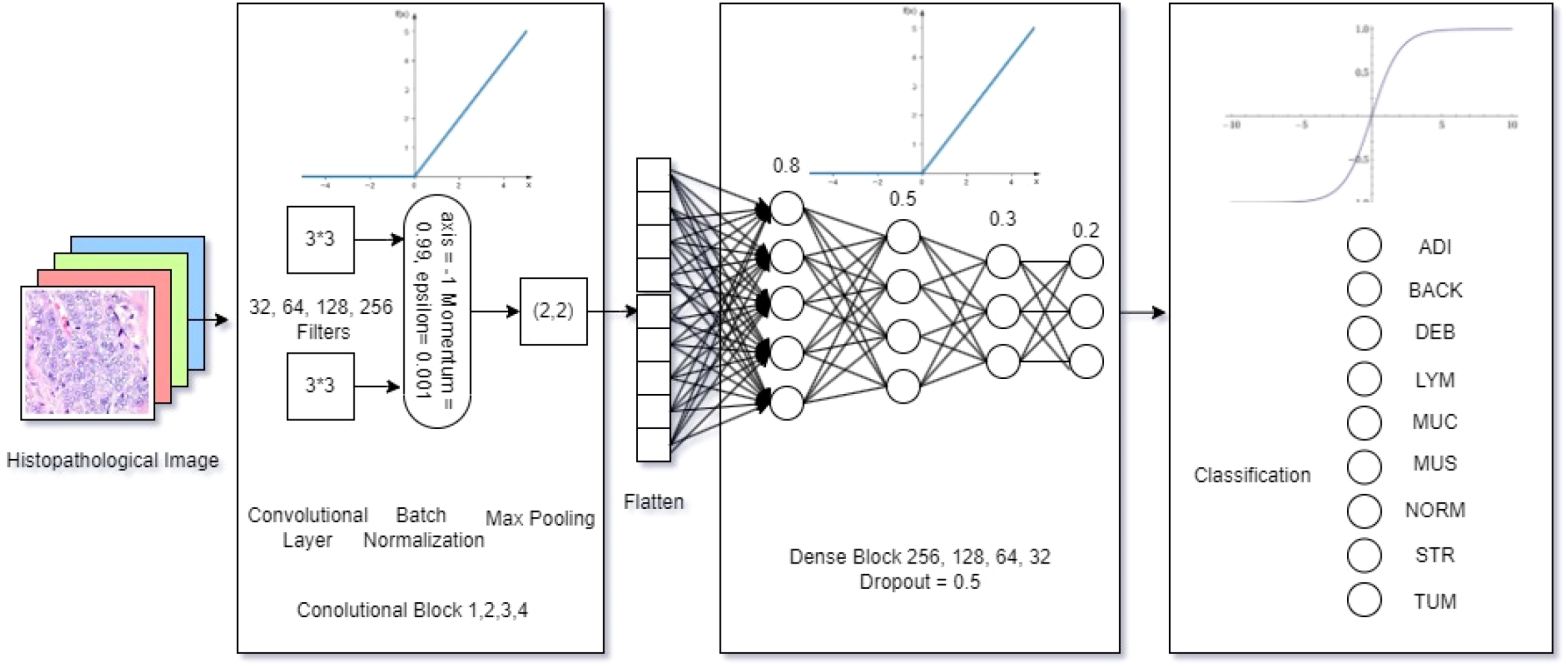
Model architecture.


The size of the model had 3,514,153 trainable and 928 non-trainable parameters within 13.46 megabytes, which defined the lightweight nature of the proposed model. As shown in **Figure 2**, an input histopathology image of 224 * 224 pixels was marked on a GRB matrix in the initial step. Then, the convolutional layer, which contains 4 individual blocks with 32, 64, 128, and 256 (3, 3) dimensioned filters, detects features in the input data using the ReLU activation function (in Equation 3), where the output of *f*(*x*) was calculated:

(3) $$f(x)=MAX(0,x)\{\begin{array}{c} x\;x\;\geq 0\;\\ 0\;x<0\; \end{array}$$

Afterward, the Batch Normalization function (in Equation 4) was adopted in normalizing the inputs to enhance the model training speed, stability, and performance, where the normalized *yi* was calculated with the mean value (*µ*)and standard deviation (*σ*) of the batch setting:


(4) $$y_{i}=\gamma (\frac{x_{i}-\mu_{b}}{\sqrt{\sigma_{b}^{2}+є}}+\beta )$$

To reduce the spatial size, the max pooling layer operates a (2, 2) dimensioned filter over the feature map. Then, the proposed model flattens the output and compares the features in a 4-block dense layer with 0.8, 0.5, 0.3, and 0.2 learning rates using the ReLU activation function (Equation 3) in the neural network. Finally, the output was multi-classified with the number of classes (*k*) as the identified categories (*i*), using the SOFTMAX (Equation 5) based on the given vector *Z*:


(5) $$\sigma {\left(Z\right)}_{i}=\frac{e^{(}Z_{i})}{\sum_{j}^{k}e^{(}Z_{j})}$$

The characterizes of the proposed model architecture shown in **Figure 1** emphasizes on its lightweight nature. The first lightweight characteristic is the depthwise convolution, which applies a single filter per input channel, which could drastically reduces the parameters needed for spatial feature extraction. Thus, this model factorization reduces computational cost and parameters by approximately a factor of the number of output channels compared to an equivalent standard convolution, and also maintains representational power.

Instead of using one or more large, dense fully-connected (FC) layers at the head of the network, a Global Average Pooling layer was designed. This layer reduces each feature map from the final convolutional block to a single value by taking the average. These values are then fed directly into the final softmax classification layer. In this case, the proposed model eliminates a massive source of parameters in FC layer and also reduces the risk of overfitting.

The model’s width (number of filters per layer) and depth (number of layers) were carefully co-designed through testing to find the smallest viable configuration that could still capture the necessary hierarchical features from the histopathology images. This avoids the common pitfall of simply stacking more layers, which leads to parameter inflation. To be summarized, there were totally 4,414,217 trainable and non-trainable parameters in a total size of 16.9 megabytes for model composition,

### 3.4 Measures

This research quantitatively evaluate the proposed lightweight model’s performance. The evaluation focused on the accuracy, categorical loss, classification report, as well as the confusion matrix. The equations Equations 6-10 (42–44) interpret the key performance metrics, including Accuracy (*Acc*), Categorical Loss (*L*), Precision (*P*), Recall (*R*), Specificity(*Sp*), F1 Score (*F*1), and Support (*S*). To all the expressions, TP stands for True Positive, TN is True Negative, FP means False Positive, and FN is False Negative. In Equation 6, *ACC* is the measurement of how well the model performs on a dataset:


(6) $$Acc=\frac{TP+TN}{TP+FP+TN+FN}$$

In Equation 7, *L* is the dissimilarity between the true label distribution ( $y_{i}$ ) and the predicted label distribution in a class (*C*)


(7) $$L=\sum_{i}^{C}y_{i}Log(y_{i}))$$

The Equation 8 presented *P* refers to the true positive predictions out of all the instances the model has predicted as positive:


(8) $$P=\frac{TP}{TP+FP}$$

In Equation 9, *R* is the sensitivity, the proportion of actual positive instances that the model correctly identified as positive:


(9) $$R=\frac{TP}{TP+FN}$$

Whereas the Specificity (*Sp*) is measured in Equation 10


(10) $$Sp=\frac{TN}{TN+FP}$$

Further in Equation 11, *F*1 refers to harmonic mean of precision and recall provide balance between them.


(11) $$F1=2*\frac{P*R}{P+R}$$

And Support(*S*) (Equation 12) is the number of actual occurrences of each class in a dataset:


(12) S=TP+FN

The above metrics established foundation for analysis results from extensive experiments. Particularly, the accuracy as the key indicator to be used to compare with benchmark studies over the past 5 years.

## 4 Data analysis

### 4.1 Data preprocessing

The **Figure 1** demonstrated the sample distribution, whereas **Table 2** listed the statistical view of the RGB color distribution of input images in the 3 datasets.

**Table 2:** Color distribution test.

Index	CRC-VALHE-7K	NCT-CRC-HE-100K	Merged
Pixel Value Mean	167.56	168.27	168.22
Blue channel	180.84	180.01	180.06
Green channel	136.51	135.94	135.98
Red channel	185.34	188.86	188.62
Pixel Value STD	44.06	43.28	43.33
Blue channel	30.88	24.09	24.60
Green channel	42.24	34.06	34.67
Red channel	30.32	24.13	24.61

The range of RGB color distribution is from 0 to 255.

In VAL-HE-7K, the MEAN (*µ*) pixel value of 167.56 indicated that the images have a moderate level of brightness. Specifically, for the blue channel, the color was recorded as 180.84, the green channel has a value of 136.51, and the red channel’s was 185.34. A standard deviation (*σ*) of 44.06 suggests a moderate degree of contrast in the images. Specifically, the blue channel had a *σ* of 30.88, the green channel was at 42.24, while the red channel was at 30.32. The *µ* pixel value of NCT-CRC-HE-100K was 168.27, indicating that the images are moderately bright compared to the previous datasets. For the blue channel, the *µ* pixel value was recorded as 180.01, the green channel has a value of 135.94, and the red channel was recorded as 188.86. A *σ* of 43.28 suggests a moderate degree of contrast in the images. In particular, the blue channel had *σ* of 24.09, the green channel’s was 34.06, and the red channel’s was 24.13. Hence, by applying the Equation 2, there were 106,987 images qualified for further model training, testing, and validation.

In the cases of NCT-CRC-HE-100K and VAL-HE-7K, the distribution color tests indicated a variance of RGB color between these 2 datasets. Thus, the use of a merged dataset could increase data variety to improve the generality of the proposed model. In this research, the merged dataset (VAL-HE-7K and NCTCRC-HE-100K) was used for model training, testing, and validation.

### 4.2 Model performance test

Model performance test recorded model learning curve of *Acc* and *L* in the training, validation, and test processes. **Table 3** displayed the results of 6 extensive experiments, which is crucial for understanding how different training setups (sample sizes, data splits, and epochs) affect the performance of a model in terms of accuracy and loss. It provides insights into the scalability of the model and the effectiveness of different data handling strategies, which is essential to optimize DL models to achieve the best possible performance on various tasks.

**Table 3:** Performance test summary.

No.	Condition	Sample size(*n*)	Epoch (*e*)	*ACC*	*L*
1	CRC-7K	7,180	13	0.879	0.074
				0.854	0.082
				0.868	0.080
2	NCT-100K	100,000	10	0.974	0.020
				0.967	0.025
				0.969	0.023
3	NCT-100K/CRC-7K	107,180	10	0.959	0.026
				0.932	0.046
				0.931	0.048
4	NCT-100K/CRC-7K	106,987	10	0.960	0.030
	Outlier Removed			0.953	0.037
				0.951	0.037
5	NCT-100K/CRC-7K	106,987	100	0.999	0.001
	Outlier Removed			0.988	0.051
				0.987	0.059
6	NCT-100K/CRC-7K	106,987	100	0.999	0.000
	Outlier Removed			0.989	0.074
	Optimization			0.990	0.075

The **Table 3** provides a comprehensive overview of model performance across different extensive experiments and highlights the impact of sample size, data splitting, and training epochs on accuracy and loss. demonstrates that larger sample sizes generally lead to better performance, and Experiment 6 shows the best overall results. As listed in **Table 3**, six step-by-step extensive experiments were executed using the NCT-CRC-HE100K, CRC-VAL-HE-7K, and merged dataset with the 80–20 rule applied in order to test the performance of the model under different data cleaning and splitting conditions.

Experiments 1 and 2 were tested with the original datasets, NCT-CRC-HE-100K and CRC-VAL-HE7K, respectively, whereas Experiment 3 was executed using the combined dataset. Experiments 4 and 5 used a 99% Gaussian distribution (N = 106,987) in 10 and 100 epochs, respectively. Lastly, Experiment 6 was fine-tuned in 100 epochs with data augmentation and hyperparameter fine-tuning strategy by Python Optuna to stabilize the learning process of the model.

#### 4.2.1 Experiment 1 - CRC-VAL-HE-7K

**Figure 3:**
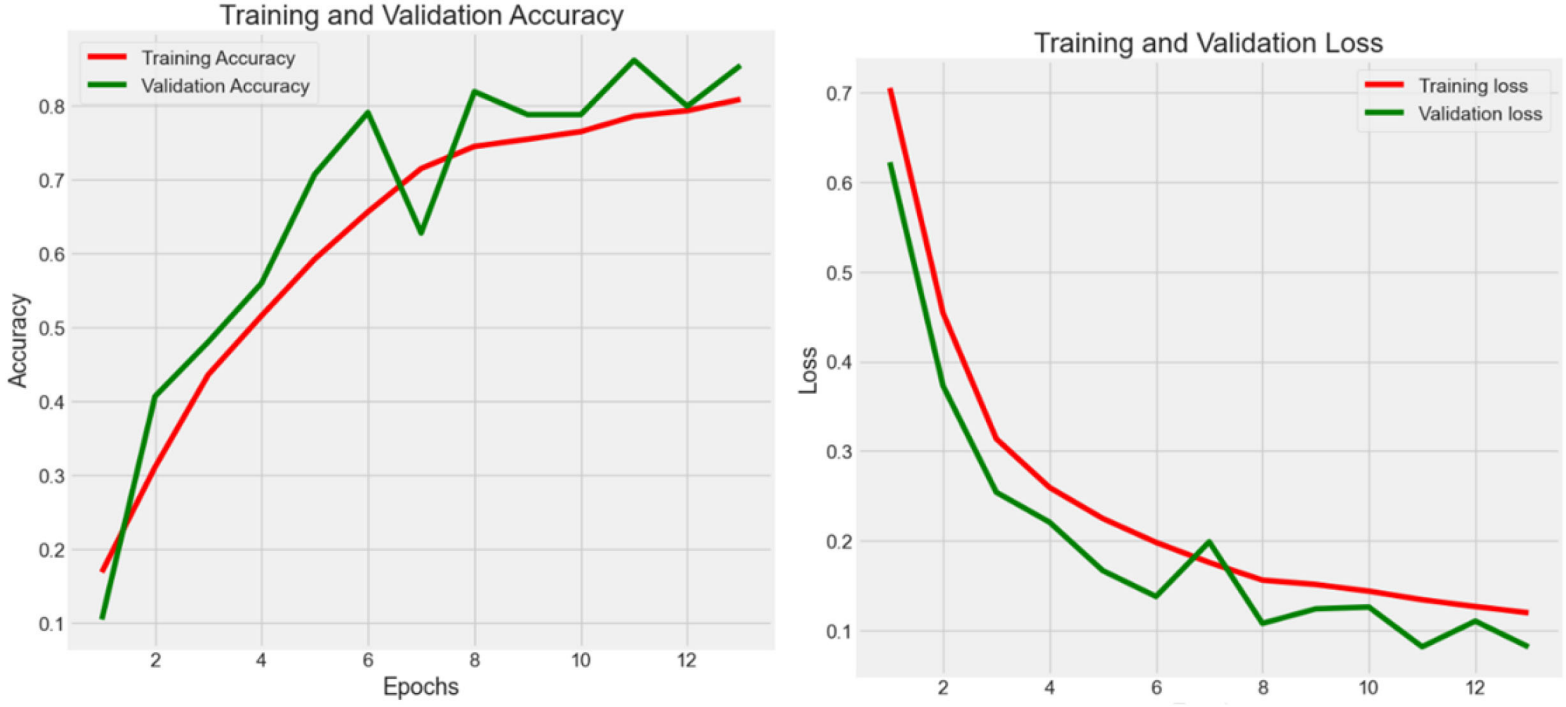
Accuracy (*Acc*) and categorical loss (*L*) of experiment 1.

Experiment 1 (**Figure 3**) had 7,180 raw data from CRC-VAL-HE-7K in the data frame while following the 80–20 rule with 13 epochs. From the result, the accuracy was 0.879, 0.854, and 0.868 in the train, validation, and test data, respectively, whereas the loss values were 0.074, 0.082, and 0.080. The testing accuracy was 0.868 with a loss of 0.148, indicating that the results did not vary significantly but could be improved with more epochs. Thus, both training and validation loss started high and decreased sharply as the number of epochs increased. The training loss decreased more smoothly and consistently compared to the validation loss, which stabilized after initial fluctuations.

#### 4.2.2 Experiment 2 -NCT-CRC-HE-100K

**Figure 4:**
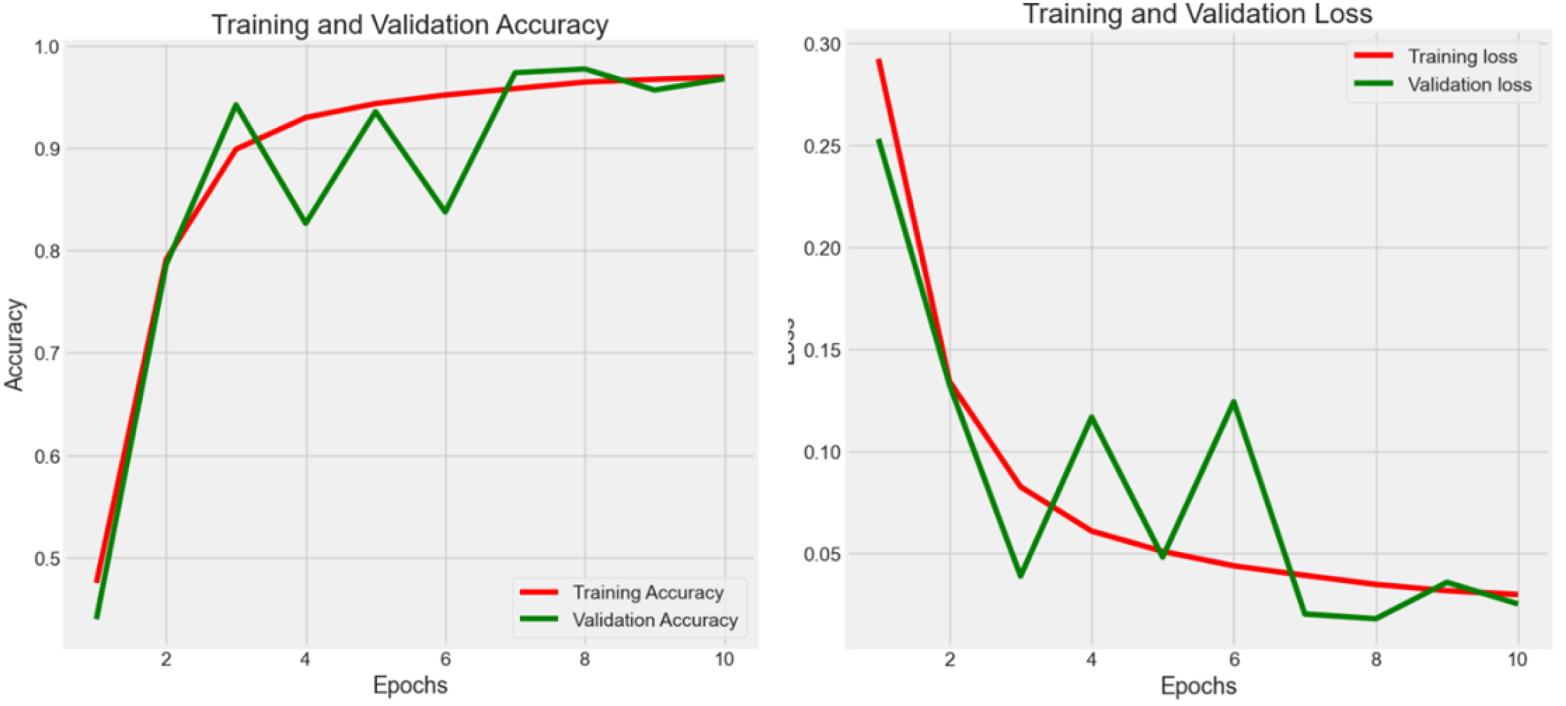
Accuracy (*Acc*) and categorical loss (*L*) of experiment 2.

Experiment 2 (**Figure 4**) had 100,000 raw data from NCT-CRC-HE-100K in the data frame while following the 80–20 rule with 10 epochs. The accuracy was 0.974, 0.967, and 0.969 in the train, validation, and test datasets, respectively, whereas the loss values were 0.020, 0.025, and 0.023. The testing accuracy was 0.969 with a loss of 0.023, indicating that the model performed well but could be further improved with more data and epochs, as well as by applying noise-decreasing approaches. The validation loss, in particular, showed sharp spikes, which could indicate issues with model stability or the presence of outliers in the data affecting the model’s performance.

Across Experiments 1 and 2, there is a common trend of initial improvements in both loss and accuracy as the number of epochs increases. However, the presence of fluctuations, especially in validation metrics, suggests challenges to the stability of the model, the possibility of overfitting, and the sensitivity to the validation dataset. These insights could guide further refinement of the model, such as adjusting model complexity, implementing regularization techniques, or revising data preprocessing and augmentation strategies to enhance model robustness and generalization.

#### 4.2.3 Experiment 3 - cross-dataset validation

**Figure 5:**
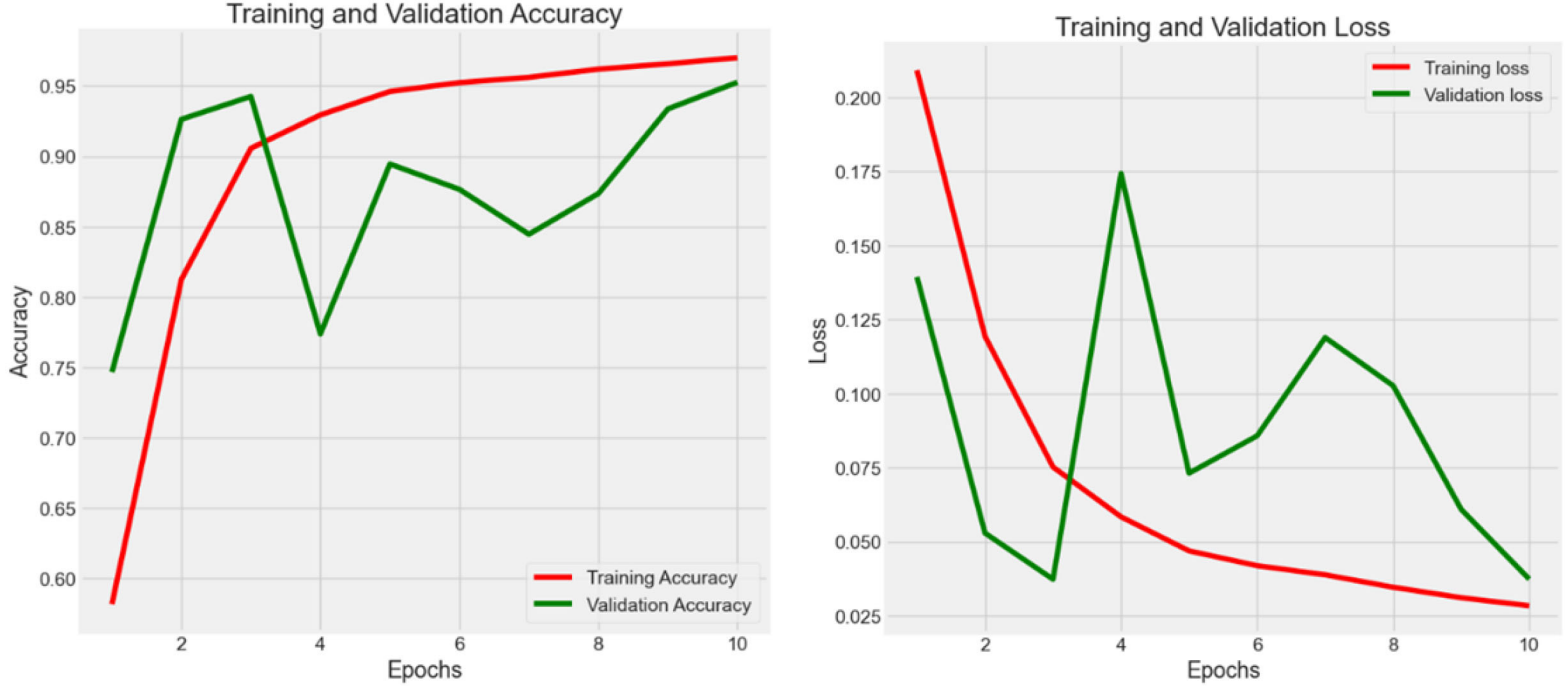
Accuracy (*Acc*) and categorical loss (*L*) of experiment 3.

In Experiment 3 (**Figure 5**), the model was trained using the combined dataset, consisting of 107,180 samples from CRC-VAL-HE-7K and NCT-CRC-HE-100K. The training process spanned 10 epochs, resulting in a training accuracy of 0.959 with a loss of 0.026. The validation and testing accuracies were slightly lower, at 0.932 and 0.931, respectively, with corresponding losses of 0.046 and 0.048. This experiment showed the most volatility among the six. The validation loss had significant spikes, particularly around epochs 2, 6, and 8. The training loss decreased more steadily. The results across the training, validation, and testing phases did not vary significantly, suggesting that the outlier removal technique contributed to more consistent performance. Lastly, the performance drop compared to Experiments 2, result of lower accuracy and higher categorical loss in Experiment 3 suggest that the combination of samples from different datasets had introduced noise, which is expected due to the domain shift between datasets. However, the model maintains high accuracy. This demonstrates a strong degree of robustness and suggests that the features learned by the lightweight CNN are generalizable across different sample preparations within the same broader data source.

#### 4.2.4 Experiment 4 - parametric cleaning

**Figure 6:**
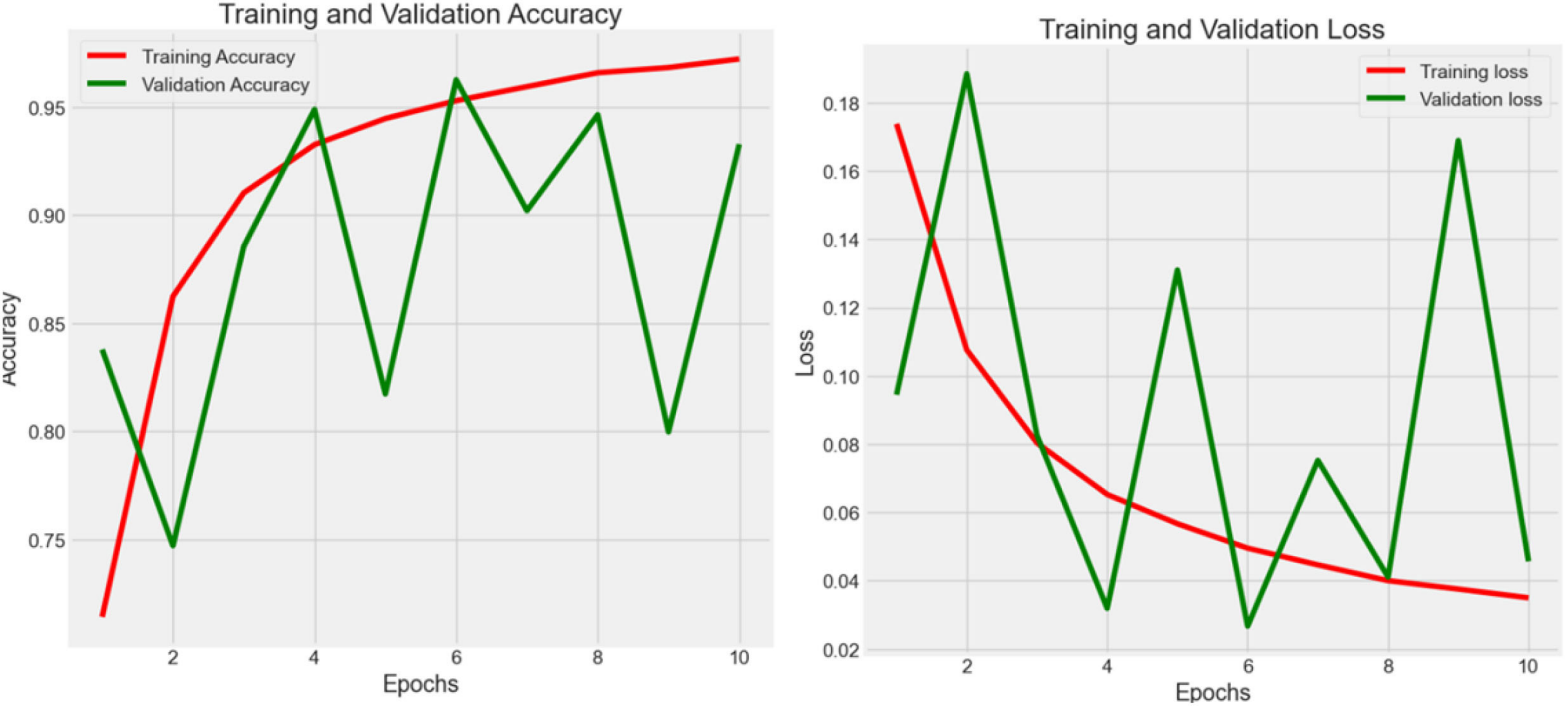
Accuracy (*Acc*) and categorical loss (*L*) of experiment 4.

In Experiment 4 (**Figure 6**), the model was trained on the same datasets, but with 106,987 rows after removing 1% of outliers using the 99% normal distribution rule. The training spanned 10 epochs, resulting in a training accuracy of 0.960 and a loss of 0.030. Validation and testing accuracies were 0.953 and 0.951, with the same loss value of 0.037. The training loss starts high and decreased steadily over epochs. The validation loss showed more fluctuation but generally decreases, with a notable spike around epoch 4. The performance improvement compared to Experiment 3 suggests that the use of a parametric cleaning approach improves model performance in the classification tasks. After merging the data sets, feature learning becomes challenging due to the complexity of feature extraction. In this case, further enhancement is necessary.

#### 4.2.5 Experiment 5 - enhancement

**Figure 7:**
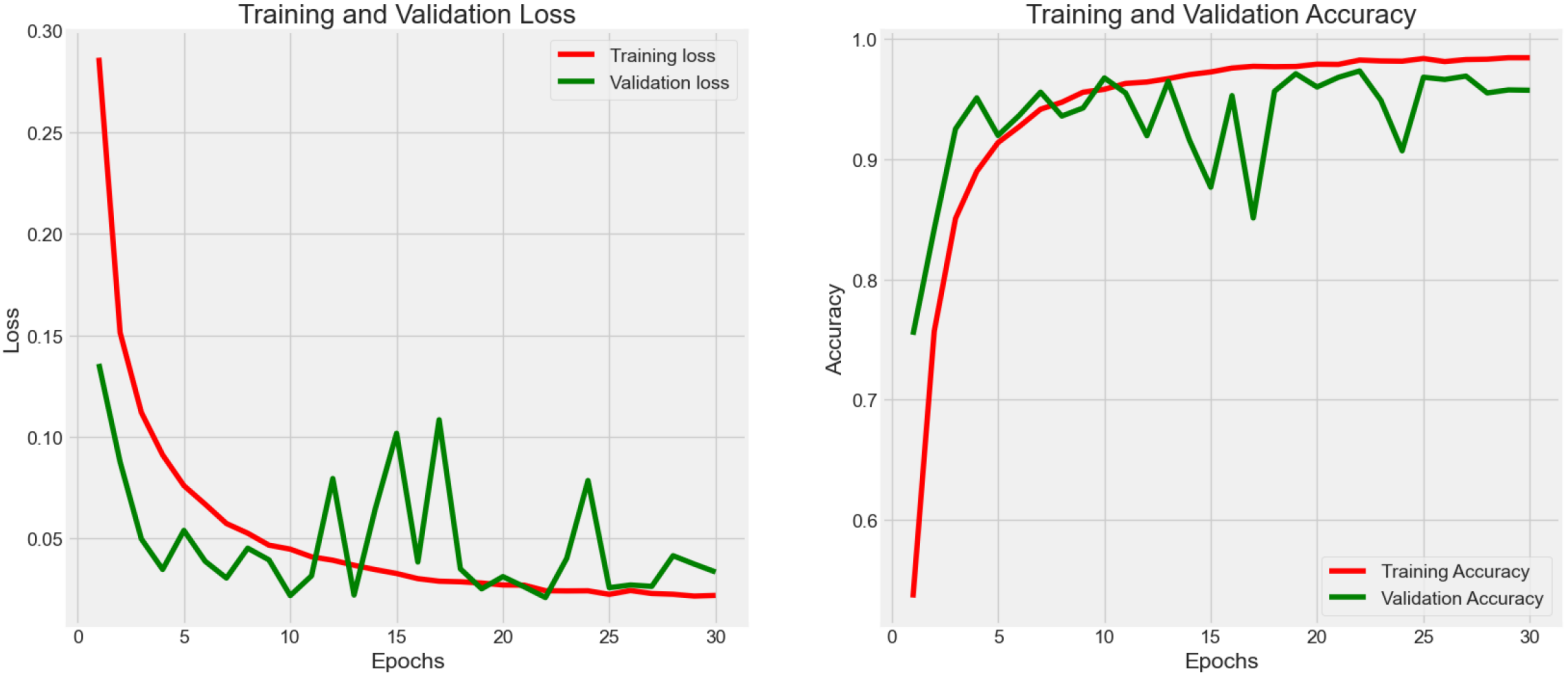
Accuracy (*Acc*) and categorical loss (*L*) of experiment 5.

In **Figure 7**, the authors set the number of epochs to 100 to train, validate, and test the model in order to boost the effectiveness of learning. The cleaned dataset was registered at 106,987 rows after using the 99% normal distribution rule. As a result, the accuracy reached 0.987 with 0.059 loss.

#### 4.2.6 Experiment 6 - augmentation and hyperparameter fine-tuning

**Figure 8:**
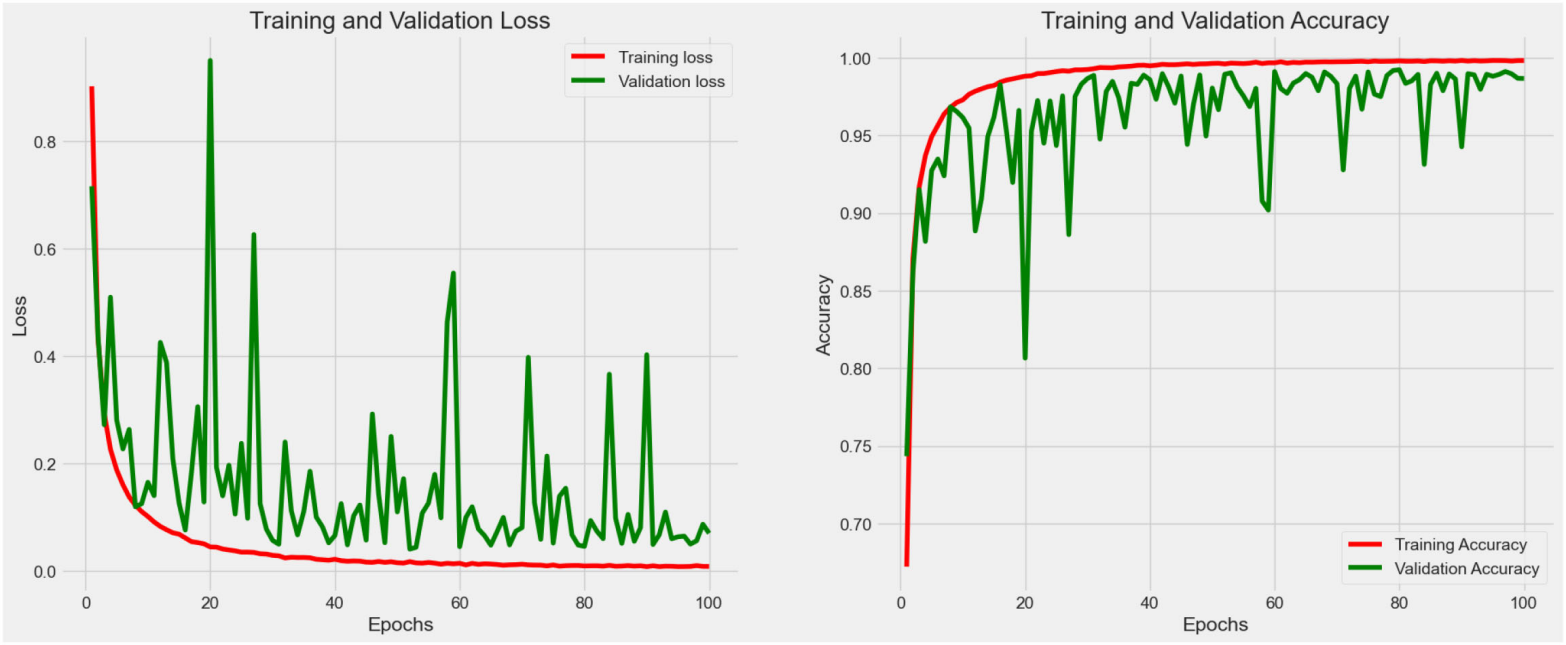
Accuracy (*Acc*) and categorical loss (*L*) of experiment 6.

As shown in **Figure 8**, fluctuation in the training process was observed in Experiment 5. In this case, an optimization strategy (data augmentation and hyperparameter fine-tuning) was applied to improve the stability as well as the learning efficiency of the proposed model. After fine-tuning, the model consumes 16.9 megabytes with 4,414,217 total parameters. As a result, the accuracy reached 0.990 with 0.075 loss in the testing process. The augmented data had allowed the weakly-parameterized CNN model to train on a more diversified set of images. This helps the model generalize better to unseen images in future clinical context, as reflected in the high accuracy (0.990) reported after hyperparameter fine-tuning. For our proposed model, we performed 5-fold cross-validation with accuracy as 0.990 ± 0.003 (mean ± 95% CI) with a 95% confidence interval (CI) of [0.987, 0.993].

### 4.3 Classification report

**Figure 9:**
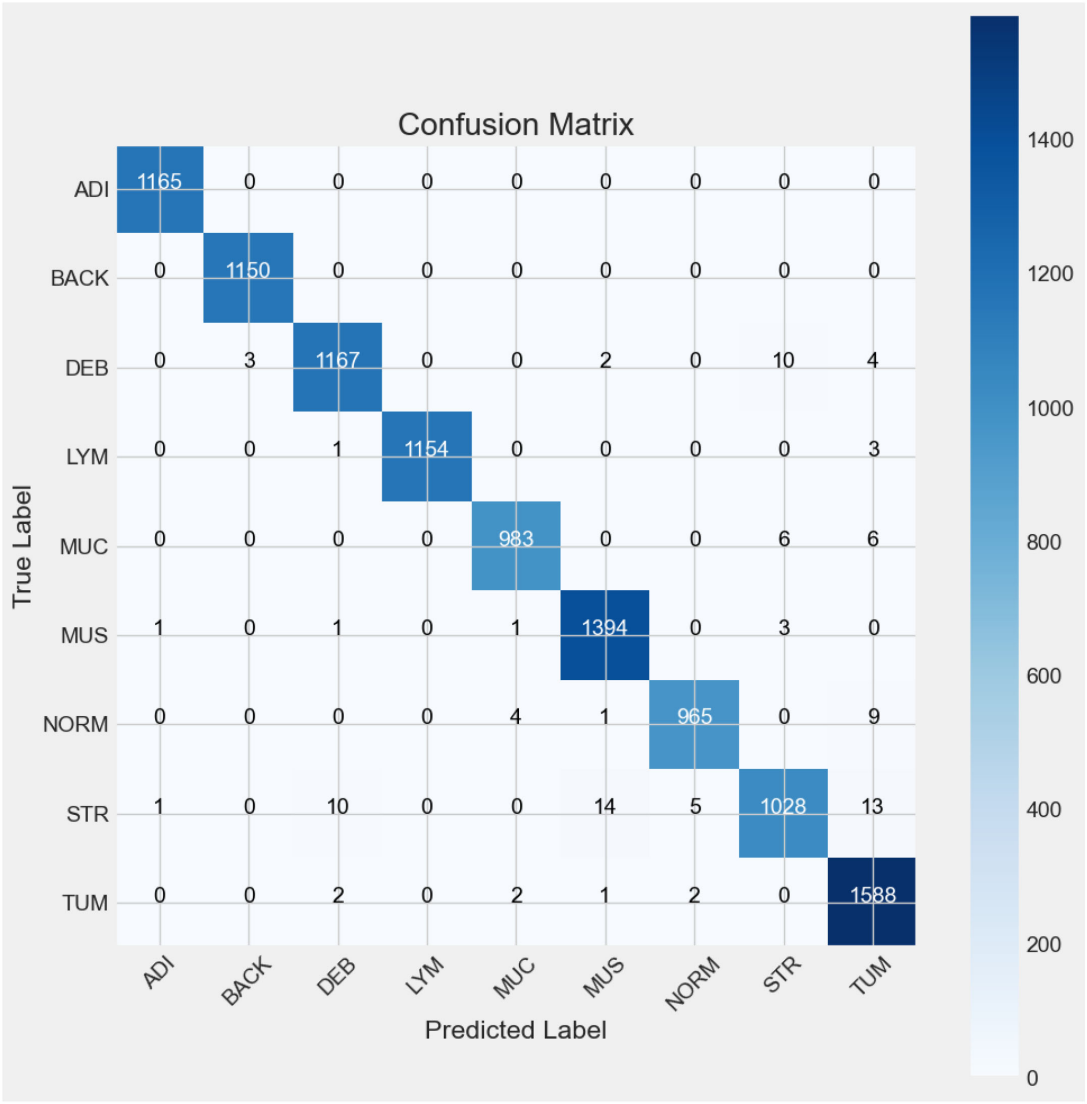
Confusion matrix.

The classification report (**Table 4**) presents a detailed confusion matrix that illustrates not only the classification performance through correctly predicted instances (true positives) but also the misclassification behavior of the model. In statistical terms, a confusion matrix (**Figure 9**) is a 9 ∗ 9 table, where each row represents the actual classes (true labels) and each column represents the predicted classes by the model.

**Table 4:** Classification report.

	Precision	Sensitivity (recall)	Specificity	F1 score	Support
ADI	1.00	1.00	1.00	1.00	1,165
BACK	1.00	1.00	1.00	1.00	1,150
DEB	0.99	0.98	0.99	0.99	1,186
LYM	1.00	1.00	1.00	1.00	1,158
MUC	0.99	0.99	1.00	0.99	995
MUS	0.99	1.00	1.00	0.99	1,400
NORM	0.99	0.99	1.00	0.99	979
STR	0.98	0.96	0.98	0.97	1,071
TUM	0.98	1.00	0.99	0.99	1,595
Accuracy				0.99	10,699
Macro Avg	0.99	0.99	0.99	0.99	10,699
Weighted Avg	0.99	0.99	0.99	0.99	10,699

For a model classifying nine categories (ADI, BACK, DEB, LYM, MUC, MUS, NORM, STR, and TUM), each diagonal category contains the number of observations that have been correctly classified for that particular category. The confusion matrix above (see **Figure 6**) indicates the representativeness of the classification model’s performance with the test dataset. There were nine categories identified from Experiment 5, and for each of the categories, most predictions hit on the target’s true labels. In the ADI category, all the 1165 samples were correctly predicted. Similarly, all the BACK samples were accurately predicted. In the case of DEB, there were 1167 samples predicted as they were labeled, but 3 were predicted as BACK, 2 as MUS, 4 as TUM, and 10 as STR. LYM predictions were spot on 1154 out of 1158 instances, with 1 and 3 misclassifications to DEB and TUM, respectively. MUC had 983 out of 995 successful predictions with 6 mislabeled instances each to STR and TUM. The MUS category was correctly predicted 1394 out of 1400 instances with 1 misclassification each to ADI, DEB, and MUC, and 3 misclassified instances to STR. In the case of NORM, there were 965 out of 979 successful predictions with 1 misclassification to MUS, 4 to MUC, and 9 to TUM. Model predictions correctly interpreted 1028 out of 1071 STR instances with 10 misclassified instances to DEB, 14 to MUS, 5 to NORM, and 13 to TUM. Lastly, there were 1588 out of 1595 instances of TUM that were correctly classified, with 1 instance of misclassification to MUS and 2 misclassifications each to DEB, MUC, and NORM.

The **Table 4** above summarizes the classification report, including precision, recall, F1-score, and the number of supports from Experiment 6 using the proposed model. The precision values indicate that 100% of the ADI, BACK, and LYM tissues in the sample images were successfully predicted by the proposed model and matched actual observations. This is followed by precision values indicating that 99% of the predicted DEB, MUC, MUC, and NORM tissues, as well as 98% of the STR and TUM tissues, matched actual observations. Recall, also known as sensitivity, is a statistical measure that quantifies the proportion of correctly predicted positive cases out of the total number of true-positive instances in a dataset. In this case, the recall values explain the sensitivity of the proposed model in accurately identifying the true positives among the 9 data classes. Lastly, the F1 score explains the balance of precision and recall. From **Figure 9**, it showed that ADI, BACK and LYM had the highest F1 scores, at a value of 1, for the predictions among all the nine categories. DEB, MUC, MUS, NORM, and TUM have F1 scores of 0.99, followed by STR with an F1 score of 0.97. In summary, the proposed model demonstrates 99% accuracy in both macro and weighted average metrics.

### 4.4 Result visualization

**Figure 10:**
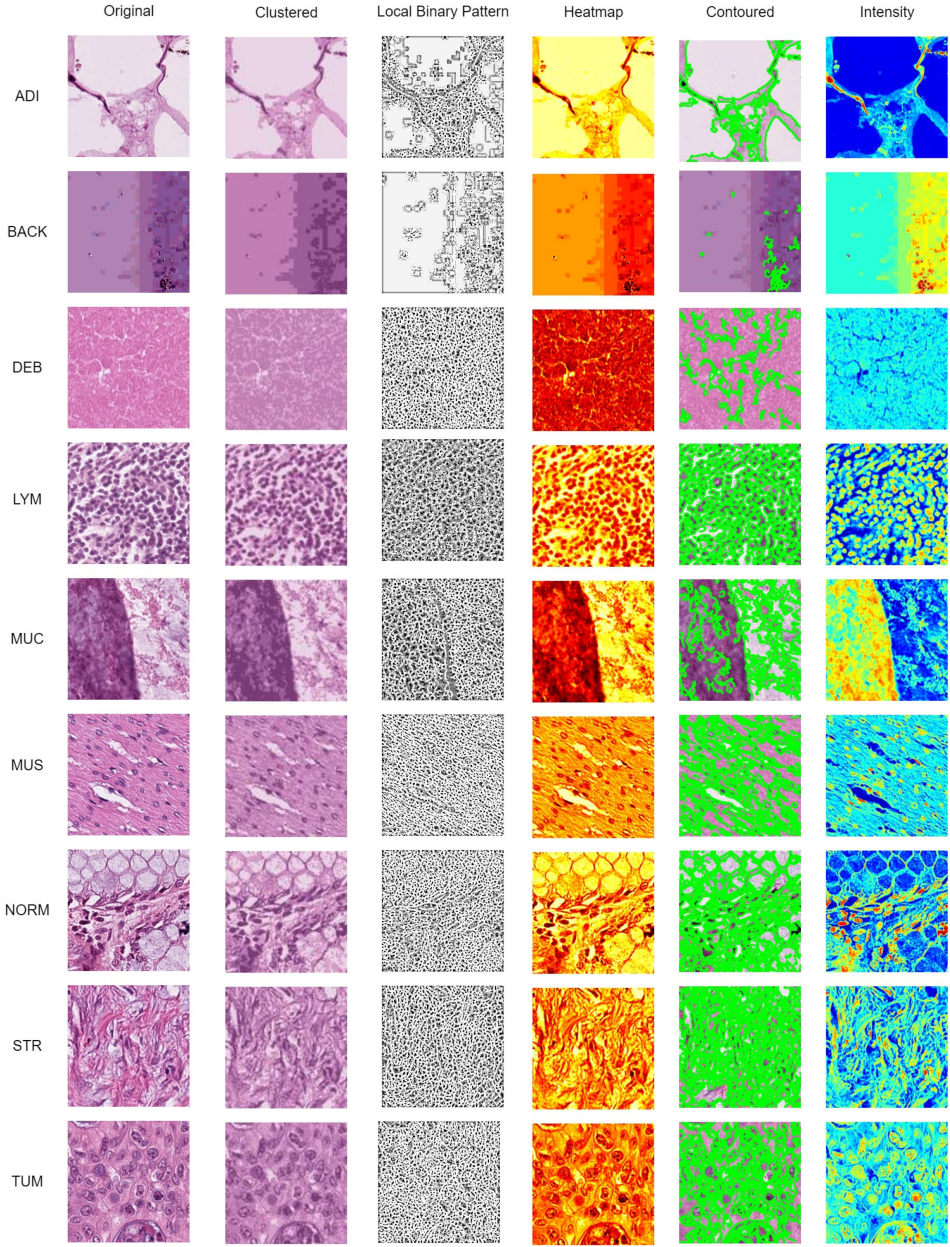
Data visualization using clustered, LBP, heatmap, contoured, and intensity imaging for clinical interpretation.

The interpretability of model predictions is critical for clinical adoption. To provide comprehensive visual insights, we generate a multi-faceted visualization for each input histopathology image via Local Binary Pattern (LBP), clustering, heatmap, contoured images, and intensity are commonly used computer vision techniques for histopathology data observation. In **Figure 10**, identified histopathology images from the 9 categories were processed with computer vision techniques to improve the clinical interpretability of the classification result.

The K-Mean Clustering method was used to group distinct segments and distinct areas using K-Means clustering algorithm (with k=3 clusters chosen empirically to represent key tissue structures). The algorithm operates on the color features of the image in the RGB space, grouping pixels into distinct regions based on color similarity. A uniform rotation-invariant LBP descriptor was used to describe the texture features of each categorical image. The LBP image highlights texture patterns and edges, which are critical for identifying histological structures.

Afterward, a heatmap was used to visualize the areas of frequency to identify concentrations using gradient-weighted class activation mapping (Grad-CAM). These gradients were globally average-pooled to obtain neuron importance weights, which were then used to create a weighted combination of the activation maps for transfer learning purpose. Contour lines were then extracted from this smoothed heatmap using the Moore-Neighbor tracing algorithm on a binary thresholded version of the map. These contours represent the boundaries of high-confidence regions identified by the model and are superimposed on the original image to delineate areas of pathological interest. Lastly, a per-pixel grayscale intensity transformation was applied, and it interpreted the color and brightness of the histopathology images from each category. The intensity view helps pathologists assess tissue staining density and cellularity without the potential bias of color variation (e.g., from H&E staining differences).

**Figure 11:**
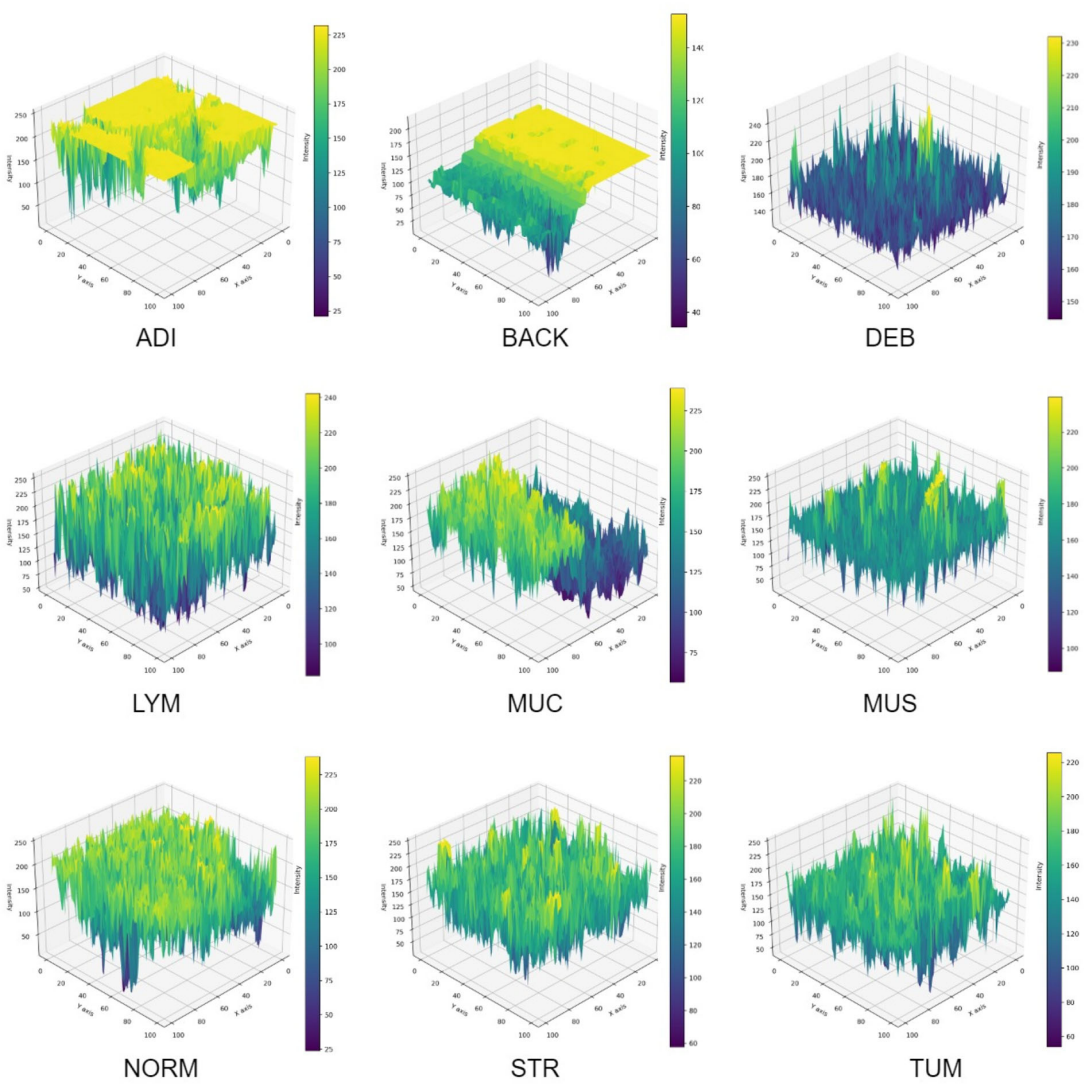
3D intensity of sample images.

In **Figure 11**, a 3D reconstruction map demonstrates the reconstruction of the spatial arrangement and depth of objects from different angles in the 2D histopathology input images. From the observation of the sample images in a human sense, there were straightforward differences in color, shape, edge, and intensity among CC tissue categories. To provide an intuitive representation of tissue morphology and density, the 3D intensity surface plots were generated for each sample image across all nine tissue classes. These plots visualize the spatial distribution of pixel intensity (brightness) across the image, effectively mapping the topographic features of the histopathology sample.

For each RGB image, we first converted it to grayscale using the luminance formula, which weights the color channels according to human perceptual sensitivity. The grayscale image represents the intensity values at each pixel location (*x,y*). Then, grayscale intensity values were treated as height values in a 3D space. Thus, the 2D image grid (*x,y*) was transformed into a 3D surface where the z-axis represents the pixel intensity. This creates a topographic map where brighter regions (e.g., nuclei, mucin) appear as peaks, and darker regions (e.g., stroma, background) appear as valleys. The 3D surface was rendered using a bicubic interpolation to smooth the surface and enhance visualization of trends. The colormap was applied to the z-values (intensity) to provide an additional visual cue for height variations. In summary, the 3D intensity plot allows pathologists to quickly assess cellularity, texture, and structural patterns.

## 5 Discussion

### 5.1 Benchmark comparison

To compare the proposed model in this study with the benchmarks, **Table 5** below lists the overview of benchmark studies over the past 5 years with precision values ranging from 0.869 to 0.997 using various CNN architectures with different histopathology datasets.

**Table 5:** Benchmark comparison.

Architecture	Dataset	ACC	Author & year
GS-PCANet	LC25000	0.909	Ram et al. (27)
Ensemble of adapted CNNs	LC25000	0.972	Salmi and Rustam (45)
MobileNetV2	12,500 Histopathology	0.997	Tasnim et al. (10)
ALEXNET	LC25000	0.970	Hamida et al. (46)
ResNet-18, 30 and 50	CRAG dataset	0.939	Çakmak and Pacal (2)
CNN+Swim Transform	NCT-CRC-HE-100K	0.937	Qin et al. (47)
Multi-scale gradient GAN	NCT-CRC-HE-100K	0.869	Jiang et al. (28)
Color-CADx	NCT-CRC-HE-100K	0.993	Sharkas and Attallah (36)
CRCCN-Net	NCT-CRC-HE-100K	0.992	Kumar et al. (29)
dResNet and DeepSVM	NCT-CRC-HE-100K	0.997	Fadafen and Rezaee (8)
Inception V3	NCT-CRC-HE-100K	0.974	Anju and Vimala (9)
Ensemble learning CNN	NCT-CRC-HE-100K	0.961	Ghosh et al. (22)
ResNet 50	NCT-CRC-HE-100K	0.993	Tsai and Tao (24)
ResMLP+SwimTran+Xception	NCT-CRC-HE-100K	0.999	Pacal and Attallah (33)
TransNetV	NCT-CRC-HE-100K CRC-VAL-HE-7K	0.985	Tanveer et al. (48)
Ensemble CNN	NCT-CRC-HE-100K CRC-VAL-HE-7K	0.961	Ghosh et al. (22)
ResNet-50	NCT-CRC-HE-100K CRC-VAL-HE-7K	0.977	Shawesh and Chen (23)
VGG-16,-19, InceptionV3, ResNet-50	NCT-CRC-HE-100K CRC-VAL-HE-7K	0.988	Intissar and Yassine (49)
CNNReFeatureBlock	NCT-CRC-HE-100K CRC-VAL-HE-7K	0.991	Firildak et al. (50)
Lightweight CNN	NCT-CRC-HE-100K CRC-VAL-HE-7K	0.990	Proposed Model

The comparison considered accuracy value as the most important metric for model performance. Over the past 5 years, histopathology image classification regarding colon cancer was accomplished by researchers worldwide. Tasnim et al. (10) applied MobileNet V2 and achieved 0.997 accuracy in their experiment with 12,500 histopathology images.

Models trained exclusively on the NCT-CRC-HE-100K dataset demonstrate a wide performance range (Accuracy: 0.869 – 0.999), with the highest accuracy (0.999) achieved by a sophisticated hybrid ResMLP+SwimTran+Xception architecture (33). This is closely followed by models like dResNet+DeepSVM and Color-CADx, which also achieve top-tier performance (≥ 0.993). Studies by Tsai and Tao (24) Kumar et al. (29) Sharkas and Attallah (36), and Fadafen and Rezaee (8) gained model accuracy surpassing 0.990. This variance underscores that architectural innovation remains a primary driver of high accuracy on a single, well-curated dataset.

In contrast, models evaluated on the combined NCT-CRC-HE-100K and CRC-VAL-HE-7K dataset present a more rigorous test of generalizability, as they must perform well on images from a separate validation set. Performance in this group is strong and more clustered (Accuracy: 0.961 – 0.991).

The proposed Lightweight CNN model achieves an accuracy of 0.990 within this cohort, performing competitively against other contemporary models like CNNReFeatureBlock (0.991) (50) and a multi-model ensemble (49) with 0.988 accuracy.

As the comparison result, the current research improved the use of CNN in CC histopathology image classification with superior accuracy performance and a lightweight nature. The current research combined the NCT-CRC-HE-100K and CRCVAL-HE-7K datasets and applied a parametric data cleaning process to improve model learning performance.

### 5.2 Pathological interpretive visualization

In this study, the visualizations presented in **Figures 10**, **11** were specifically designed to bridge the gap between the extraction of computational features and the morphological interpretative framework used by pathologists. The K-Mean Clustering approach applied as data-centric visualization aligns with the pathologist’s initial, low-power assessment of architectural patterns, which provides an objective, quantitative basis for tissue segmentation, which can be used to isolate specific regions for further quantitative analysis (e.g. measuring stromal percentage) (51). The algorithm automatically partitions the image into structurally coherent regions (e.g., epithelial clusters, stromal bands, luminal spaces) (52) to simulate the way a pathologist mentally segments tissue to organize analysis.

Texture is a critical differentiator in histology. The LBP visualization enhances textural patterns that are key to diagnosis. The uniform patterns highlighted by LBP correspond to repetitive structure of normal glandular epithelium (NORM) (53), disordered texture of tumor glands (TUM) (54), and fibrous texture of stromal tissue (55). The LBP provides a computational evidence for validation by pathologist gains subjectively at high power with an objective measure of tissue disorder.

The Grad-CAM Heatmaps and Contoured visualizations, which are model-centric interpretative visualization methods, reinforce the pathologist’s fundamental reasoning of predicted result, and function as an automated highlighting tool for less experienced practitioners or high-volume workloads. The activation of the model consistently localizes to areas showing pools of extracellular MUC (56), glandular architecture (57), nuclear hyperchromasia (58) of TUM, and desmoplastic STR reaction (59). The regions highlighted by the Grad-CAM heatmap directly correspond to morphological features of diagnostic significance (59, 60).

While Grad-CAM highlights where the model looked, the 3D intensity plot helps explain what the model perceived in those regions. The 3D intensity translates the 2D slide into a 3D topographic map that correlates directly with cellularity and density. The 3D visualization quantifies the subjective assessment of cellular density. The 3D surface is a direct measure of textural heterogeneity. Stromal tissue (STR) might have a moderately rough texture, while homogeneous mucin (MUC) appears as a smooth plateau. A TUM image would show numerous, irregular peaks, while an ADI image would show large valleys with sparse, isolated peaks (representing adipocyte nuclei). The use of 3D intensity plots quantitatively assesses the size, shape and separation of the tumor (61). To be summarized, the above-mentionedd approaches form a multi-modal explanation system in terms of a collaborative human-AI decision-making process.

### 5.3 Key findings and implications

From the experiment results, the following findings can be stated. First of all, the experimental results illustrated that a merged dataset, a rigorous data cleaning approach, and targeted fine-tuning could lead to improvement on image classification performance, thereby contributing to faster and more reliable diagnostic processes with the proposed lightweight CNN model. Second, the fine-tuning process produced a significant enhancement, demonstrating that the lightweight CNN is not only efficient but also highly accurate for the classification of histopathology images in colon cancer. Third, the improved stability and performance metrics demonstrate the model’s potential for deployment in resource-constrained environments such as mobile diagnostic devices. Lastly, the detailed classification report reinforces the model’s robustness across various tissue classes, which is critical for achieving precision in diagnostic applications.

The steep improvement in both training and validation metrics during the Experiment 6 (see **Table 4**; **Figure 8**) indicated that the model quickly learns the most salient features of the dataset. This rapid convergence suggests that the proposed architecture and hyperparameters are well-suited to the task. However, it also implies that fine-tuning efforts should focus on the underlying pattern recognition where improvements on feature extraction become more subtle and challenging. The growing gap between training and validation performance, particularly in accuracy, suggests a tendency towards overfitting. This implies that fine-tuning efforts should prioritize techniques that enhance the model’s generalization capabilities without sacrificing its ability to learn from the training data.

In summary, while the CNN model shows promising performance, the results highlight several areas where fine-tuning could lead to improvements. By carefully addressing overfitting tendencies, optimizing the learning rate strategy, refining the architecture, and stabilizing validation performance, it may be possible to develop a model that not only achieves high accuracy but also demonstrates more consistent and generalizable performance across both training and validation datasets. The fine-tuning process should be iterative, with each adjustment carefully monitored for its impact on both training and validation metrics.

### 5.4 Clinical deployment

The proposed model can be deployed as a pre-screening tool on pathologists’ workstations with rapid analysis on digitized slides by flagging regions suspicious for carcinoma (TUM, MUC) for expert review. In this case, the proposed model could significantly reduce the pathologist’s workload, decrease turnaround times, and minimize screening fatigue, especially in high-volume laboratories.

To be integrated into a hospital’s digital pathology system, the model could serve as an always-available “second reader”. After a pathologist renders a diagnosis, the system could automatically process the slide and provide a concordance check or highlight potential areas of disagreement for further scrutiny in terms of diagnostic confidence.

The lightweight nature of proposed CNN model offers distinct advantages for deployment in hospital and laboratory environments, which often have heterogeneous computing infrastructure. Specifically speaking, the deployment on front-end devices majorly reguires for processor. On a standard CPU (Intel Xeon), the model processes over 50 images per second. This speed increases to over 1,200 images per second on a high-end GPU (NVIDIA 4060Ti) and even higher if a higher GPU version or NPUs were applied, facilitating the batch processing of large datasets. The entire model requires less than 50 MB of RAM for inference (without the operating system and image data). This minimal memory requirement allows it to run concurrently with other essential hospital software without contention for resources.

## 6 Conclusion

In conclusion, this research achieved all of the objectives. First of all, the proposed lightweight CNN model (16.9 megabytes) offers a highly efficient and accurate approach to colon cancer histopathology image classification, particularly suited for environments with limited computational resources. Second, this paper realized histopathology image segmentation by constructing clustering, LBP, heatmap, contoured, and 3D views for interpretability improvement in the CC diagnosis process. Third, from the results, the proposed model gained 0.990 accuracy with 0.075 loss in performance metrics. On the other hand, in the classification report, the proposed model achieved a macro average precision of 0.99, a recall (sensitivity) of 0.99, specificity of 0.99, an F1 score of 0.99, and a total of 10,699 support instances. By integrating a data cleaning strategy based on parametric Gaussian distribution, the model effectively enhances data quality, leading to superior classification performance. The study’s findings highlight the model’s competitive edge over existing benchmarks, demonstrating its potential as a reliable diagnostic tool.

The study proposes that a lightweight CNN is a robust and efficient solution for histopathology image classification, providing a promising foundation for further developments in this field. These findings contribute to the development of efficient and accurate CC histopathology image processing systems, particularly in resource-constrained environments. The lightweight model design and data cleaning strategy can serve as a foundation for future research in this area.

The primary limitation is the potential for inherent bias in the NCT-CRC-HE-100K and CRC-VAL-HE-7K datasets, while being large-scale public benchmarks, are sourced from a single institution. The cross-dataset validation and data augmentation is the steps towards assessing generalizability and bias mitigation, but the definitive test requires validation on a fully external, multi-centric dataset. Therefore, a key direction for future work is to acquire and validate our model on such an external dataset comprising images from multiple, geographically diverse medical centers. Secondly, future research can be built upon this work by exploring further transfer learning for classification tasks in multi-organ histopathology to enhance generalization. This work lays the groundwork for developing advanced, resource-efficient AI solutions in medical diagnostics with streamlined deployment on mobile and edge devices.

## Data Availability

The original contributions presented in the study are included in the article/supplementary material. Further inquiries can be directed to the corresponding author.
